# Species distribution modeling of northern sea otters (*Enhydra lutris kenyoni*) in a data‐limited ecosystem

**DOI:** 10.1002/ece3.11118

**Published:** 2024-03-07

**Authors:** Elizabeth L. Hasan, Kristen B. Gorman, Heather A. Coletti, Brenda Konar

**Affiliations:** ^1^ College of Fisheries and Ocean Sciences University of Alaska Fairbanks Fairbanks Alaska USA; ^2^ National Park Service, Southwest Alaska Inventory and Monitoring Network Anchorage Alaska USA

**Keywords:** data‐limited, *Enhydra lutris kenyoni*, habitat mapping, MaxEnt, sea otter, species distribution modeling

## Abstract

Species distribution models (SDMs) are used to map and predict the geographic distributions of animals based on environmental covariates. Typically, SDMs require high‐resolution habitat data and time series information on animal locations. For data‐limited regions, defined as having scarce habitat or animal survey data, modeling is more challenging, often failing to incorporate important environmental attributes. For example, for sea otters (*Enhydra lutris*), a federally protected keystone species with variable population trends across the species' range, predictive modeling of distributions has been successfully conducted in areas with robust sea otter population and habitat data. We used open‐access data and employed a presence‐only model, maximum entropy (MaxEnt), to investigate subtidal habitat associations (substrate and algal cover, bathymetry, and rugosity) of northern sea otters (*E. lutris kenyoni*) for a data‐limited ecosystem, represented by Kachemak Bay, Alaska. Habitat association results corroborated previous findings regarding the importance of bathymetry and understory kelp as predictors of sea otter presence. Novel associations were detected as filamentous algae and shell litter were positively and negatively associated with northern sea otter presence, respectively, advancing existing knowledge of sea otter benthic habitat associations useful for predicting habitat suitability. This study provides a quantitative framework for conducting species distribution modeling with limited temporal and spatial animal distribution and abundance data. Utilizing drop camera information, our novel approach allowed for a better understanding of habitat requirements for a stable northern sea otter population, including bathymetry, understory kelp, and filamentous algae as positive predictors of sea otter presence in Kachemak Bay, Alaska.

## Graphical abstract

For data‐limited regions with scarce habitat or animal survey data, species distribution modeling is challenging, often failing to incorporate important environmental attributes. Here, we used open‐access data and employed a presence‐only model, maximum entropy (MaxEnt), to investigate subtidal habitat associations (substrate and algal cover, bathymetry, and rugosity) of northern sea otters (*Enhydra lutris kenyoni*) for a data‐limited ecosystem. This study provides a quantitative framework for conducting species distribution modeling with limited temporal and spatial animal distribution and abundance data and utilized drop camera information as a novel approach to better understand habitat requirements for a stable northern sea otter population.

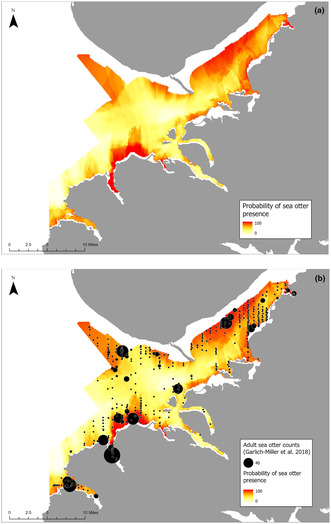

## 1 INTRODUCTION

Quantifying habitat associations and predictive modeling of animal distributions are commonly conducted using species distribution models (SDMs). Typically, SDMs rely on spatially explicit environmental information and known locations of species occurrences to construct mathematical relationships among these variables to model and predict a species' habitat use across a landscape (Drake et al., 2006; Elith et al., 2006; Guisan et al., 2002). There are various types of SDMs, including simple regression‐based methods such as generalized linear models and generalized additive models, as well as more recently developed approaches based on machine learning methods such as random forests, boosted regression trees, support vector machines, and maximum entropy. A variety of ecological questions can be addressed with SDMs, many of which have important implications for resource management. Species distribution models have commonly been used in the terrestrial realm (Robinson et al., 2011, 2017). However, in the marine realm, SDMs can be used to guide the designation and management goals of Marine Protected Areas (MPAs), which critically rely on a quantitative understanding of spatial habitat use by key species (Hameed et al., 2017). Additionally, SDMs can be used for modeling habitat suitability for invasive species (Srivastava et al., 2019), predicting species' range shifts in response to climate change (Zhang et al., 2020), and understanding the spatial ecology of infectious diseases (Slatculescu et al., 2020). Further, SDMs can be used to anticipate regions where wildlife might have an increased risk of human interactions due to industrial development or vessel activity (Blondin et al., 2020). Many of these models require species presence/absence data. To predict potential species distributions, SDMs have been shown to perform better for modeling probability of occurrence than abundance (Lee‐Yaw et al., 2022). By predicting species occurrence based on habitat suitability, we can gain a better understanding of our changing environment and how to best manage ecosystems and species, especially for data‐limited ecosystems.

Marine systems are understudied worldwide with <10% of our global oceans mapped. Multibeam sonar data are ideal for marine‐focused SDMs due to the continuous spatial coverage and systematic characterization of substrate and algal cover (Monk et al., 2010). However, for many remote regions of the ocean, multibeam data are often not available due to accessibility and technical challenges. For example, Alaska's coastline makes up approximately 50% of the national coastline for the United States, but as of 2021 only 31% of Alaska's coastal waters have been mapped with single or multibeam sonar for bathymetry (Alaska Coastal Mapping Strategy Implementation Plan 2020–2030, 2022). Thus, habitat characterization of Alaska's coastal regions is particularly challenging. In the absence of sonar data, systematic drop camera surveys can serve as a replacement. Drop cameras have been widely used as non‐invasive tools for measuring underwater habitat and organismal distributions when multibeam sonar data are not available (Bethoney & Stokesbury, 2018; Easton et al., 2015).

In addition to the environmental data noted above, modeling relationships between habitat and species occurrence across space and time (Elith & Leathwick, 2009) requires known locations for a given species of interest. Often, animals are equipped with Global Positioning System (GPS) tags or collars to track their movements and determine their locations in time and space. Advances in animal tracking technology now allow for high‐resolution GPS tracking (e.g., Hart et al., 2020; Kuhn et al., 2009). Sea otters (*Enhydra lutris*), the focal species of this study, are challenging to track because GPS tags cannot be affixed to the animal's pelage due to risk of hypothermia if the fur is disturbed by GPS attachment (Davis et al., 2019). Very high frequency (VHF) radio tags have been deployed on sea otters, but this method requires surgery to implant the device, these tags do not remotely transmit data, and thus, all data must be collected in the field using telemetry receivers and is therefore often cost limiting (Davis et al., 2019; Garshelis & Siniff, 1983). The current method for estimating sea otter population abundance entails aerial surveys, generally during summer months, where an observer records the location and abundance of observed animals for a particular day and time (Bodkin & Udevitz, 1999).

Understanding habitat associations for current sea otter distributions and potential future distributions is important for a variety of reasons. First, sea otters have experienced a long history of population change after they were nearly extirpated by the commercial fur harvest between the 1700's and 1900's (Kenyon, 1969). Sea otter populations have been recovering and expanding since they were protected in 1911 (Kenyon, 1969), and in response to translocation efforts (Jameson et al., 1982). Research on population expansion and habitat suitability is paramount for understanding ecosystem correlates of current or future sea otter presence (Davis et al., 2019), particularly since sea otters are a keystone species playing a critical role in coastal marine ecosystem structure and function (Estes & Palmisano, 1974). For example, sea otters function as an ecosystem engineer across the North Pacific, including rocky (Estes et al., 1998), soft sediment (Kvitek et al., 1992), and seagrass (Foster et al., 2021; Hughes et al., 2013) communities. Second, changes in the presence or absence of sea otters may impact local resource availability both for commercial and subsistence purposes (Carswell et al., 2015; Hoyt, 2015). Commercial and personal use fisheries and mariculture can be impacted by sea otter presence (Bodkin et al., 2002; Carswell et al., 2015). Lastly, federal and state permitting of anthropogenic activities, such as oil and gas development, require consideration of impacts to marine mammals, including sea otters. Thus, it is important to accurately predict how ecosystems may change under future climate scenarios, industrial development, or other anthropogenic stressors in the context of sea otter presence in an ecosystem.

Predicting ecosystem change requires an understanding of current habitat associations and future species distribution. Sea otters use nearshore systems that are associated with canopy‐forming kelp, shallow depths, or preferred benthic prey (i.e., clams, crabs, etc.) (Gilkinson et al., 2011; Kenyon, 1969; Miller, 1974; Ribic, 1982; Rotterman & Simon‐Jackson, 1988), but finer scale subtidal habitat use, such as subtidal algal cover, substrate composition, or bathymetric complexity, has not been fully explored. Finer scale habitat attributes such as these are of interest as these features may increase predictability of sea otter species distribution models.

Northern sea otter (*E. lutris kenyoni*, hereafter sea otter) distributions have been previously modeled in Prince William Sound, Alaska (Coletti, 2006), southeast Alaska (Eisaguirre et al., 2021; Lu et al., 2020; Tinker et al., 2019; Williams et al., 2019), Washington state (Hale et al., 2022; Laidre et al., 2002), and California (Tinker et al., 2021). Recent modeling efforts have employed Bayesian state‐space models (Hale et al., 2022; Tinker et al., 2019) and diffusion models (Eisaguirre et al., 2021; Lu et al., 2020; Williams et al., 2019), frameworks that are possible due to the high resolution of sea otter abundance time series data. Calculating population dynamics requires annual estimates of changes in abundance and range (Eisaguirre et al., 2021; Hale et al., 2022; Lu et al., 2020; Tinker et al., 2019, 2021; Williams et al., 2019) because sea otter populations are structured on small scales (Davis et al., 2019). Additionally, animal movement patterns, collected via telemetry, are necessary for modeling dispersal (Hale et al., 2022; Tinker et al., 2019, 2021). However, not all sea otter populations have been surveyed with such frequent temporal coverage. For example, some populations, such as Cook Inlet, Alaska, have only been surveyed once in over a decade (Garlich‐Miller et al., 2018).

For minimally surveyed populations, the maximum entropy (MaxEnt) model (Elith et al., 2011; Phillips et al., 2006; Phillips & Dudík, 2008) is an appropriate quantitative framework as MaxEnt uniquely uses presence‐only data compared to other SDM methods such as generalized additive models (Guisan et al., 2002), support vector machines (Drake et al., 2006), and random forests (Evans et al., 2011). MaxEnt performs better than other SDMs in the model's predictive ability due to its incorporation of the lasso penalty by default (Renner & Warton, 2013); however, the lasso penalty can be incorporated into other modeling frameworks to achieve similar predictive performance. In addition, MaxEnt is resilient to overfitting (Elith et al., 2006; Valavi et al., 2022), can handle small sample sizes (Kaky et al., 2020; Kaky & Gilbert, 2016; Wisz et al., 2008), and can model complex relationships between response and predictor variables (Elith et al., 2006). We examined the role of specific substrate and algal habitat attributes, including several that have yet to be considered, as predictors of sea otter abundance in Kachemak Bay, Alaska. Including numerous substrate and algal habitat attributes that characterize the species' environment as covariates can result in a highly dimensional model, which can often be overfit. Thus, assessing performance of models with varying complexity was critical in our study of novel benthic associations.

Other than MaxEnt, SDMs often assume that species occurrences across the region of interest are representative of prevalence, but this is not always the case (Elith et al., 2011). Sea otters are highly mobile and forage underwater; therefore, it cannot be assumed that locations without sea otter observations are true absences. However, modeling presence‐only in a colonized area still retains the signal of absence, because if a habitat is unsuitable for an animal there will be no presence records (Elith et al., 2011). The MaxEnt model instead uses pseudoabsences (i.e., available space) along with occurrences to model habitat use. Here, we evaluated whether the distribution of sea otters in Kachemak Bay, Alaska can be modeled with only 2 years of habitat information and 1 year of sea otter occurrence information using MaxEnt. We also compared sea otter habitat associations for the data‐limited ecosystem of Kachemak Bay with other regions that are more data‐rich, defined as having a longer time series or fine spatial resolution, to better understand possible generalizations between sea otters and their habitat attributes. This study leveraged open‐access data to demonstrate how the MaxEnt framework may be applied to other ecosystems and the ecologically relevant questions we can answer with data that are publicly available.

## 2 METHODS

### 2.1 Study site

**FIGURE 1:**
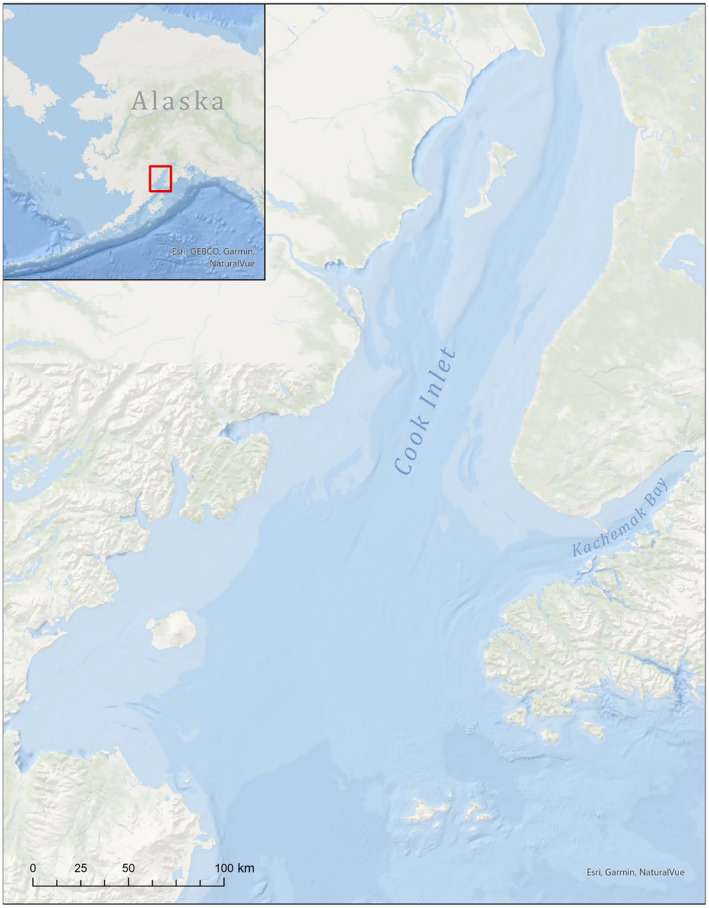
Map of Cook Inlet, located in southcentral, Alaska. Kachemak Bay is located on the eastern side of Cook Inlet.

**FIGURE 2:**
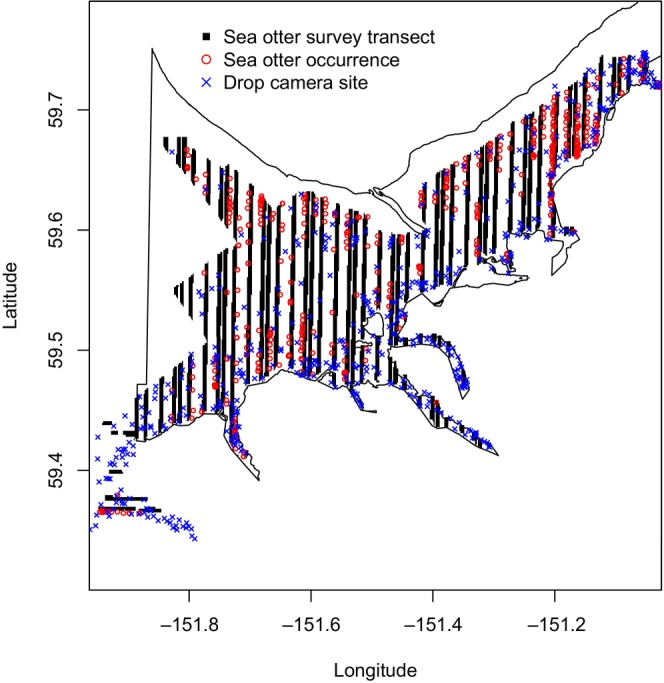
Map of the Kachemak Bay, Alaska, study site. The thin black line represents the outline of Kachemak Bay. The thick black lines indicate surveyed areas for sea otter abundance in 2017. Red circles (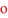) indicate northern sea otter sightings and blue crosses (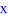) indicate drop camera sites.

This research was conducted in Kachemak Bay, Alaska (Figures 1 and 2) – a highly productive, estuarine ecosystem located in southcentral Alaska that connects to Cook Inlet at the eastern coastline and supports significant macroalgal, invertebrate, fish, and marine mammal populations. Sea otters have inhabited Kachemak Bay since the 1970's, and the current population is thought to be stable at an estimated ~6000 adults (Esslinger et al., 2021; Garlich‐Miller et al., 2018). It was important to use a stable population for model development to understand if other uncolonized regions are suitable to support a future stable population. The distribution of sea otters has expanded from Kachemak Bay into Cook Inlet and is expanding northward further into Cook Inlet (Figure 1) (Esslinger et al., 2021; Garlich‐Miller et al., 2018).

### 2.2 Model components

The subtidal habitat data used in this study are publicly available through the National Oceanic and Atmospheric Administration (NOAA) (Field et al., 2020). In 2016 and 2017, NOAA collected 518 subtidal habitat videos in Kachemak Bay via a drop camera. The drop camera consisted of two cameras, one low‐resolution (720 pixels) camera (Sartek Industries, Inc.) and one high‐resolution (1080 pixels) GoPro camera, and two lights for visibility at depth. Only high‐resolution camera data were characterized for this study. Instruments were mounted on a stainless steel tripod frame. The drop camera was lowered to depths between 0 and 170 m with a tether and passed over the benthos for various durations of time (~2–10 min), while recording continuous video. Point locations of each survey were marked with a GPS device.

To obtain fine‐scale habitat information, average percent cover (0–100%) of each substrate type and algal group was visually determined over the duration of each high‐resolution video. Substrate utilized a modified Wentworth scale consisting of boulder, cobble, pebble, gravel, shell litter, sand, and mud (Wentworth, 1922). Algae consisted of understory kelp (Order: Laminariales), macroalgae (all macroalgae, not including Laminariales), filamentous/microalgae, and coralline algae. Raster surfaces for each habitat category were produced using ordinary kriging in ArcGIS (ArcGIS, 2012) to obtain continuous percent cover (0–100%) of each habitat attribute throughout the survey region. Raster surfaces have a cell resolution of 131 × 131 m. Bathymetry data were collected by NOAA in 2008 and 2009 (Field et al., 2020) and are represented in meters. Rugosity was derived from bathymetry using the rugosity tool in ArcGIS (ArcGIS, 2012) and is represented as a ratio of the true surface area over the geometric surface area. Multicollinearity among habitat data was assessed using a stepwise variance inflation factor method via the vif function from the usdm package in R (Naimi et al., 2014; R Core Team, 2022). Mud was excluded from the model due to high multicollinearity, resulting in 12 covariates included in the model (Table 1).

**TABLE 1:** Habitat attributes and respective units and values for covariates included in the MaxEnt model.

Habitat attribute	Units	Values
Boulder	Percent cover	0–100
Cobble	Percent cover	0–100
Pebble	Percent cover	0–100
Gravel	Percent cover	0–100
Shell litter	Percent cover	0–100
Sand	Percent cover	0–100
Understory kelp	Percent cover	0–100
Macroalgae	Percent cover	0–100
Filamentous algae	Percent cover	0–100
Coralline algae	Percent cover	0–100
Bathymetry	Depth	<0
Rugosity	Ratio	≥1

Sea otter abundance data used in this analysis were collected by the United States Fish and Wildlife Service (USFWS) in 2017 (Esslinger et al., 2021; Garlich‐Miller et al., 2018) via aerial abundance surveys flown using the method described by Bodkin and Udevitz (1999). Four replicate surveys consisting of a series of 400 m wide strip transects were flown during May 2017 in the north–south direction across Kachemak Bay, starting and ending at the 0 m tide line. Starting points were randomly selected and subsequent transects were flown every 4 km for each replicate. These strip transects cumulatively covered 40% of the total area of Kachemak Bay (Garlich‐Miller et al., 2018). Observations from eastern Cook Inlet surveys that fell within NOAA's habitat study area were also included in the analysis. For the eastern Cook Inlet surveys, a series of 400 m wide strip transects were flown in the east–west direction from the 0 to 40 m depth contour. These surveys were flown 7 km apart and replicated three times during May 2017, resulting in a 15% coverage of the total area. Data were represented with sea otter counts at each observation point recorded as geographic coordinates (Garlich‐Miller et al., 2018).

Counts were not corrected based on the intensive search unit correction factor, as described in Garlich‐Miller et al. (2018), because locations alone, not counts, were used in this analysis. Replicate surveys were combined for this study to represent all observed sea otter locations in Kachemak Bay during the 2017 survey. Only on‐transect sea otter observations were used for this analysis; off‐transect observations were discarded, as they extended beyond the 400 m transect band and were not consistently recorded. Duplicate observations at any single coordinate were not included in the model, as MaxEnt will only allow one observation per grid cell to avoid pseudoreplication. Only adult sea otter locations were used as pups are dependent on female sea otters. Polygons of survey transects were produced by creating polylines from transect start and end points (Esslinger et al., 2021). Sea otter data were constrained to the extent of habitat data.

### 2.3 MaxEnt model

A MaxEnt model (Elith et al., 2011; Phillips et al., 2006; Phillips & Dudík, 2008) was produced to determine subtidal habitat associations of sea otters and to provide a framework for predictive modeling of sea otter population expansion given spatial and temporal data limitations imposed on this study. For this, we used the MaxEnt algorithm (Elith et al., 2011; Phillips et al., 2006; Phillips & Dudík, 2008) through the ENMeval package in R (Kass et al., 2021; R Core Team, 2022). Sea otter observations collected during the 2017 aerial abundance survey (Esslinger et al., 2021; Garlich‐Miller et al., 2018) were used as presence locations in the model with only one observation retained per grid cell. A total of 391 sea otter presence locations and 10,000 background points were used. Initial sensitivity analysis did not demonstrate improved model performance with fewer (6000) or greater (14,237; maximum number of background points available due to raster resolution) background points (Renner et al., 2015). Background points were randomly selected from strip transect polygons (Figure 2) as this was considered available space of the surveyed extent. Training and testing data were separated using random k‐fold partitioning with five folds or groups. Random k‐fold partitioning randomly separates the data into k folds, or groups. Cross‐validation is conducted by leaving out one group at a time and calculating the out of sample predictive performance as the area under the receiver operating characteristic curve (AUC). The AUC values for training and testing are averaged over the five runs of cross‐validation.

Model settings were determined through model tuning in the ENMeval package, as described by Kass et al. (2021). Default MaxEnt version 3.4.3 settings were used, except for permissible types of feature classes and the regularization multiplier. Twenty models were created with varying types of feature classes (i.e., linear, hinge, and combinations of linear and quadratic, and linear, quadratic, and hinge) and regularization multipliers from 1 to 5. Feature class type dictates the shape of the relationship of covariates to response (Bohl et al., 2019). The regularization multiplier dictates the penalty for model complexity with a high regularization multiplier corresponding to a large penalty (Elith et al., 2011; Merow et al., 2013; Phillips & Dudík, 2008). Together, these settings influence model complexity. Model selection was conducted with two methods by filtering model results: a sequential method for cross‐validation that favors the lowest average test omission rate and the highest average AUC (Kass et al., 2021; Radosavljevic & Anderson, 2014), and information‐theoretic methods (Akaike's Information Criterion corrected for small sample sizes, AICc; Burnham & Anderson, 2004). Sequentially selected models were compared to AICc selected models for final optimal model selection (Velasco & González‐Salazar, 2019; Warren & Seifert, 2011).

Once the most parsimonious model was selected, model performance was evaluated by comparing results to a null model distribution. This allowed for testing the success of modeling species distributions for our data‐limited system. Some applications of MaxEnt models report model estimates, but there is no way to evaluate performance within the algorithm. Bohl et al. (2019) developed a framework for evaluating model performance by comparing the empirical model to a null model distribution. The novelty of this approach is a null model simulation that is validated with randomly selected points, which simulate occurrences, against the same testing dataset and background points used in the empirical model. This allows for more direct comparison on model performance than in previous evaluation frameworks. As a non‐parametric method, it is critical to employ an evaluation method to properly estimate effect size and model significance.

Null models were constructed over 1000 iterations by calibrating with a random sample of points within the strip transect polygons and the same background points from the empirical model. Null models were validated with the same testing dataset as the empirical model, so that performance measures were directly comparable (Bohl et al., 2019). Model performance of the empirical and null models was evaluated with the AUC for the training (AUC_train_) and testing (AUC_val_) datasets, the difference between AUC_val_ and AUC_train_ (AUC_diff_), and omission error rate (OR) with a threshold that leads to 10% omission of calibration records (Liu et al., 2005). AUC_val_ and AUC_train_ are measures of discriminatory ability and AUC_diff_ and OR are measures of overfitting (Bohl et al., 2019). Standardized effect sizes (Ulrich & Gotelli, 2010) and *p*‐values were calculated to test whether the empirical model performed better than the null model distribution (Bohl et al., 2019). Covariate percent contribution was gathered from the MaxEnt model output to determine the importance of habitat attributes in predicting sea otter presence. The empirical model was then applied to all grid cells in the study area (i.e., full extent of habitat raster layers) to predict the probability of sea otter occurrence based on relationships with habitat covariates and demonstrate capability for out of sample prediction. Sea otter survey results, including abundance of adult sea otters at each observed point (Garlich‐Miller et al., 2018), were superimposed on the map of predicted probability of occurrence to visually assess model performance.

## 3 RESULTS

### 3.1 Model components

**FIGURE 3:**
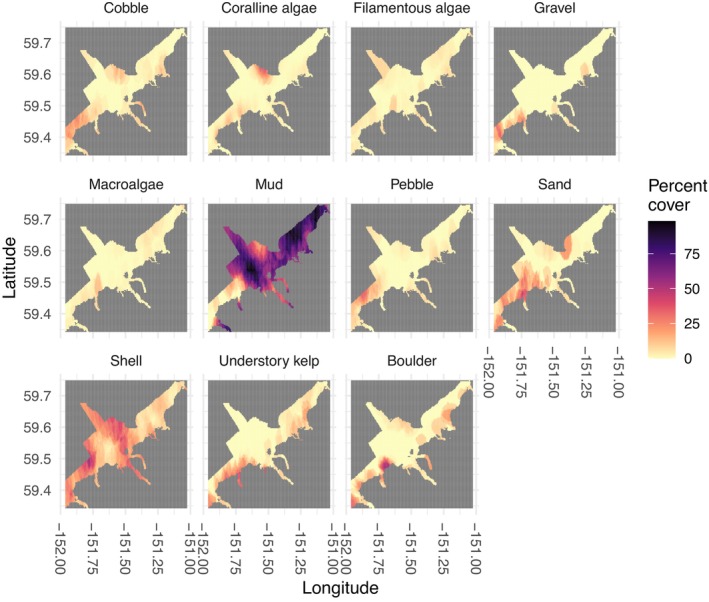
Raster surfaces for each substrate (boulder, cobble, pebble, gravel, shell litter, sand, and mud) and algal (understory kelp, macroalgae, filamentous algae, and coralline algae) attribute throughout the survey region in Kachemak Bay, Alaska. Darker colors represent higher percent cover and lighter colors represent lower percent cover.

Analysis of drop camera data revealed variation in percent cover of substrate and algal habitat attributes throughout Kachemak Bay (Figure 3). Percent cover of larger sediment grain sizes (i.e., boulder and cobble) was higher on the south side of the bay, trending toward an average of 50% cover, than the north side. Percent cover of mud was higher in the inner bay, trending toward 80% cover, while cover of pebble, gravel, shell litter, and sand was higher in the outer bay, trending toward 50% cover. Mud was the most consistently observed habitat attribute throughout Kachemak Bay. Algal cover was found primarily in shallower waters at the edges of the bay. Understory kelp was found primarily on the south side of the bay, while macroalgae and filamentous algae were variable throughout the bay. Percent cover of coralline algae was higher in the outer bay, trending toward 20% cover, than the inner bay.

### 3.2 Model performance

**FIGURE 4:**
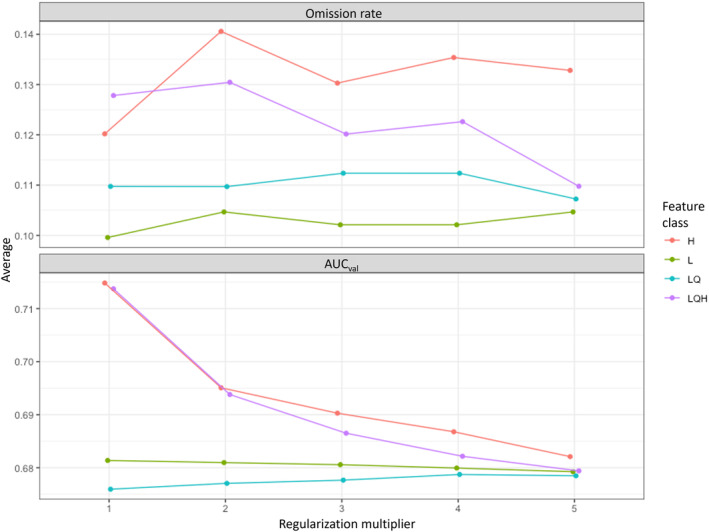
Line graphs of candidate model settings for model tuning. Lines are colored by feature settings (H, hinge; L, linear; LQ, linear and quadratic; LQH, linear, quadratic, and hinge). The *x*‐axis indicates the regularization multiplier (1–5), and the *y*‐axis indicates the evaluation metric value with omission rate on the top panel and AUC_val_ (area under the receiver operator curve for the test dataset) on the bottom panel.

The evaluation of model settings with the sequential method resulted in selection of features set to linear and the regularization multiplier set to one as optimal settings (Figure 4). The sequentially selected model resulted in an omission rate of 0.0996 and an AUC_val_ of 0.681. The AICc model selection method suggested features set to linear and quadratic and a regularization multiplier set to one, resulting in a more complex model than the sequentially selected model due to additional feature types. Comparatively, the omission rate of the AICc selected model was 0.110 and the AUC_val_ was 0.676, demonstrating a model that is more overfit than the sequentially selected model with a slightly lower discriminatory ability. The sequentially selected model with features set to linear and the regularization multiplier set to one was deemed to be optimal and is the empirical model referred to hereafter.

**FIGURE 5:**
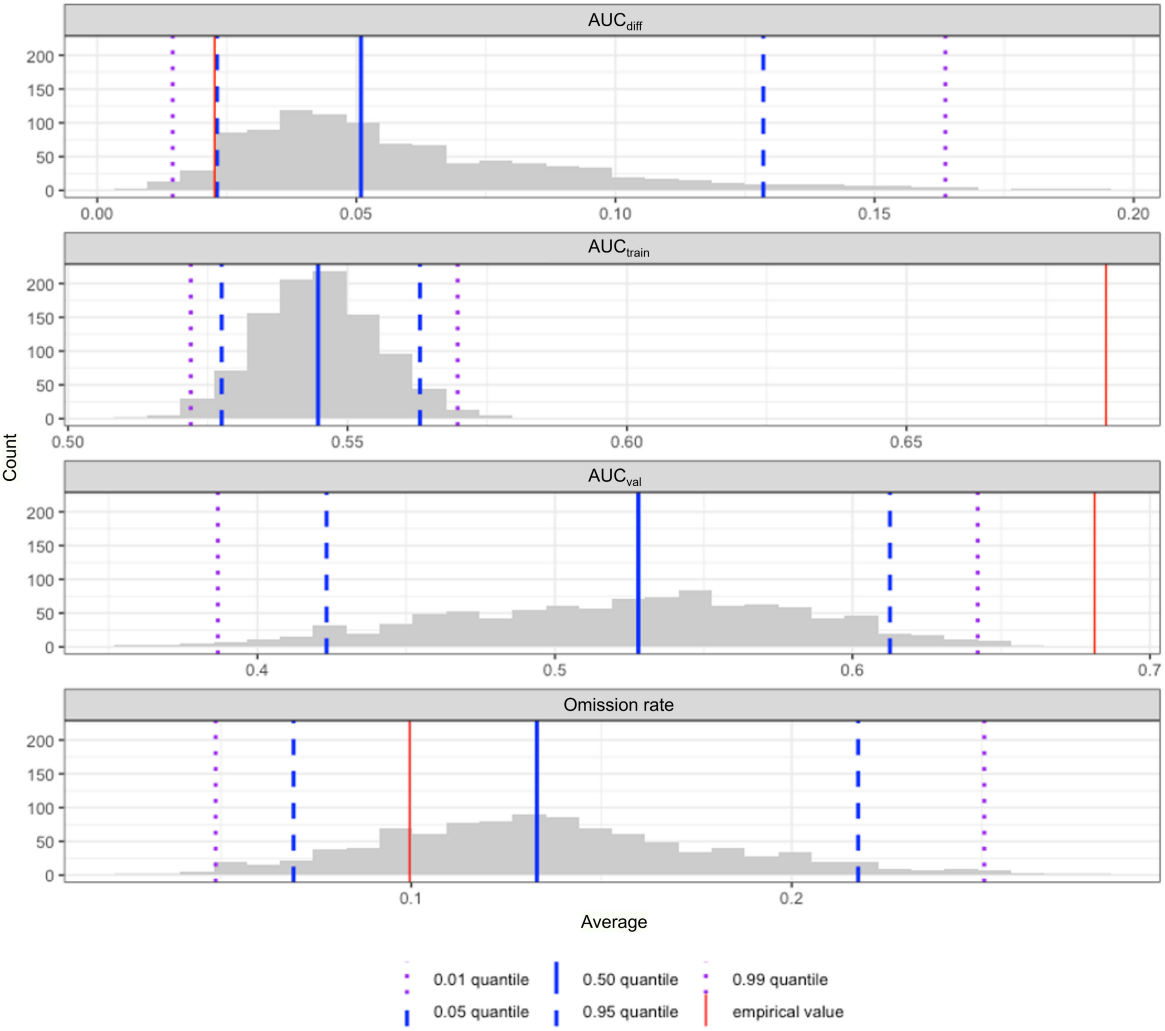
Null model distribution histograms for evaluation of MaxEnt model performance. The red line indicates evaluation metric values for the empirical model. Dashed blue lines indicate the 0.05 and 0.95 quantiles, purple indicates the 0.01 and 0.99 quantiles, and the solid blue line indicates the median of the null model distribution. The *x*‐axis of each panel corresponds to the AUC_diff_, AUC_train_, AUC_val_ (i.e., the area under the receiver operator curve for the training data, testing data, and the difference between training and testing), and omission rate value from top to bottom, respectively. The *y*‐axis indicates frequency.

The null model distribution was used to evaluate multiple model metrics against the empirical model. Discriminatory metrics, AUC_train_ and AUC_val_ for the empirical model were 0.686 and 0.681, respectively, and 0.545 and 0.524 for the null model distribution, respectively. The p‐values from a one‐sided test evaluating whether the AUC_train_ and AUC_val_ were larger for the empirical model than the null model distribution were *p* < .001 and *p* < .001, respectively (Table 2). Empirical AUC_train_ and AUC_val_ fall outside of the 99th quantile of the null model distribution, showing significantly better (*α* = 0.05) model performance than models with randomly generated presence locations in the survey area (Figure 5). Metrics of overfitting, AUC_diff_ and omission rate, for the empirical model were 0.0226 and 0.0996, respectively, and 0.0599 and 0.137 for the null model distribution, respectively. The p‐values from a one‐sided test evaluating whether the AUC_diff_ and omission rate were smaller for the empirical model than the null model distribution were *p* < .01 and *p* < .05, respectively (Table 2). Empirical AUC_diff_ and omission rate demonstrated significantly different degrees of overfitting (*α* = 0.05) from null models with randomly generated presence locations in the survey area (Figure 5, Table 2). The out of sample prediction for Kachemak Bay had 68.1% (AUC_val_) predictive accuracy with a standard deviation of 2.61% (Table 2).

**TABLE 2:** MaxEnt model evaluation metrics for the empirical model and null model distribution for sea otter habitat suitability in Kachemak Bay, Alaska, using data from 2016 to 2017.

Statistic	AUC_train_	AUC_val_	AUC_diff_	OR
Empirical mean	0.686	0.681	0.0226	0.0996
Empirical standard deviation	NA	0.0226	0.0195	0.0548
Null mean	0.545	0.524	0.0599	0.137
Null standard deviation	0.0107	0.0151	0.0130	0.0168
*Z* score	13.2	10.5	−2.86	−2.23
*p*‐value	5.14 × 10^−40^	5.13 × 10^−26^	0.00211	0.0129

Abbreviations: AUC, stands for the area under the receiver operator curve and is evaluated for training data, testing data, and the difference between training and testing; NA, not applicable; OR, omission rate.

*Note*: *Z* scores and *p*‐values compare the empirical model to the null model distribution with a one‐sided test.

### 3.3 Habitat associations

**FIGURE 6:**
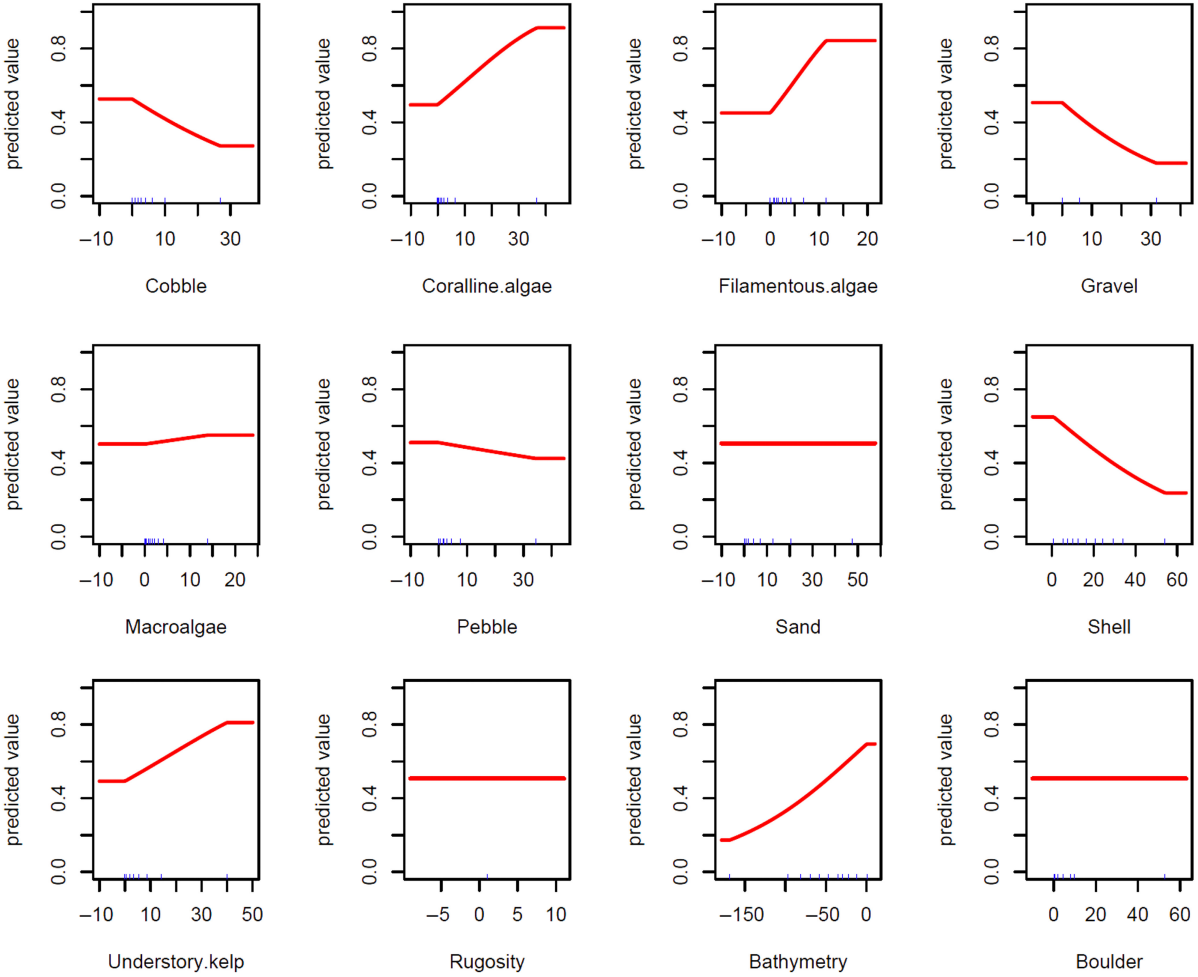
Response curves of MaxEnt model covariates with linear features and a regularization multiplier of one. The *x*‐axis indicates covariate data value in percent cover (cobble, coralline algae, filamentous algae, gravel, macroalgae, pebble, sand, shell litter, understory kelp, and boulder), depth (bathymetry), and rugosity ratio (rugosity). The *y*‐axis indicated the probability of sea otter presence based on modeled relationships with each covariate.

The optimal model resulted in bathymetry, shell litter, and filamentous algae as the three most influential habitat attributes predicting current probability of sea otter occurrence in Kachemak Bay, Alaska, with 43, 18.2, and 18% contribution, respectively (Table 3). Shallower depths and increasing percent cover of filamentous algae had positive relationships with probability of sea otter occurrence (Figure 6). Increasing percent cover of shell litter had a negative relationship with probability of sea otter occurrence (Table 3, Figure 6). Understory kelp was the next most important habitat attribute at 6.2% contribution and had a positive relationship with probability of sea otter occurrence, though there was a substantial decrease in percent contribution between filamentous algae and understory kelp (Table 3, Figure 6). Gravel, pebble, coralline algae, cobble, and macroalgae (in order of importance) had low (<6%) contributions for predicting sea otter occurrence (Table 3). Rugosity, boulder, and sand had no predictive value for sea otter occurrence. Permutational importance, which measures the decrease in training AUC when values of each variable are randomly permuted (Kalle et al., 2013), over 120 iterations also followed these trends, except for pebble, macroalgae, and sand, which had zero permutational importance (Table 3). Mud was excluded from the analysis, as it was highly correlated with other environmental covariates.

**TABLE 3:** Percent contribution, permutational importance, and relationship direction of habitat attributes in predicting sea otter presence.

Variable	Percent contribution	Permutation importance	Relationship direction
Bathymetry	43.0	25.2	Positive
Shell litter	18.2	23.4	Negative
Filamentous algae	18.0	11.9	Positive
Understory kelp	6.20	8.40	Positive
Gravel	5.30	5.80	Negative
Pebble	3.40	0	Negative
Coralline algae	2.90	13.1	Positive
Cobble	2.30	11.9	Negative
Macroalgae	0.600	0	Positive
Boulder	0	0.200	NA
Rugosity	0	0.200	NA
Sand	0	0	NA

Abbreviation: NA, not applicable.

**FIGURE 7:**
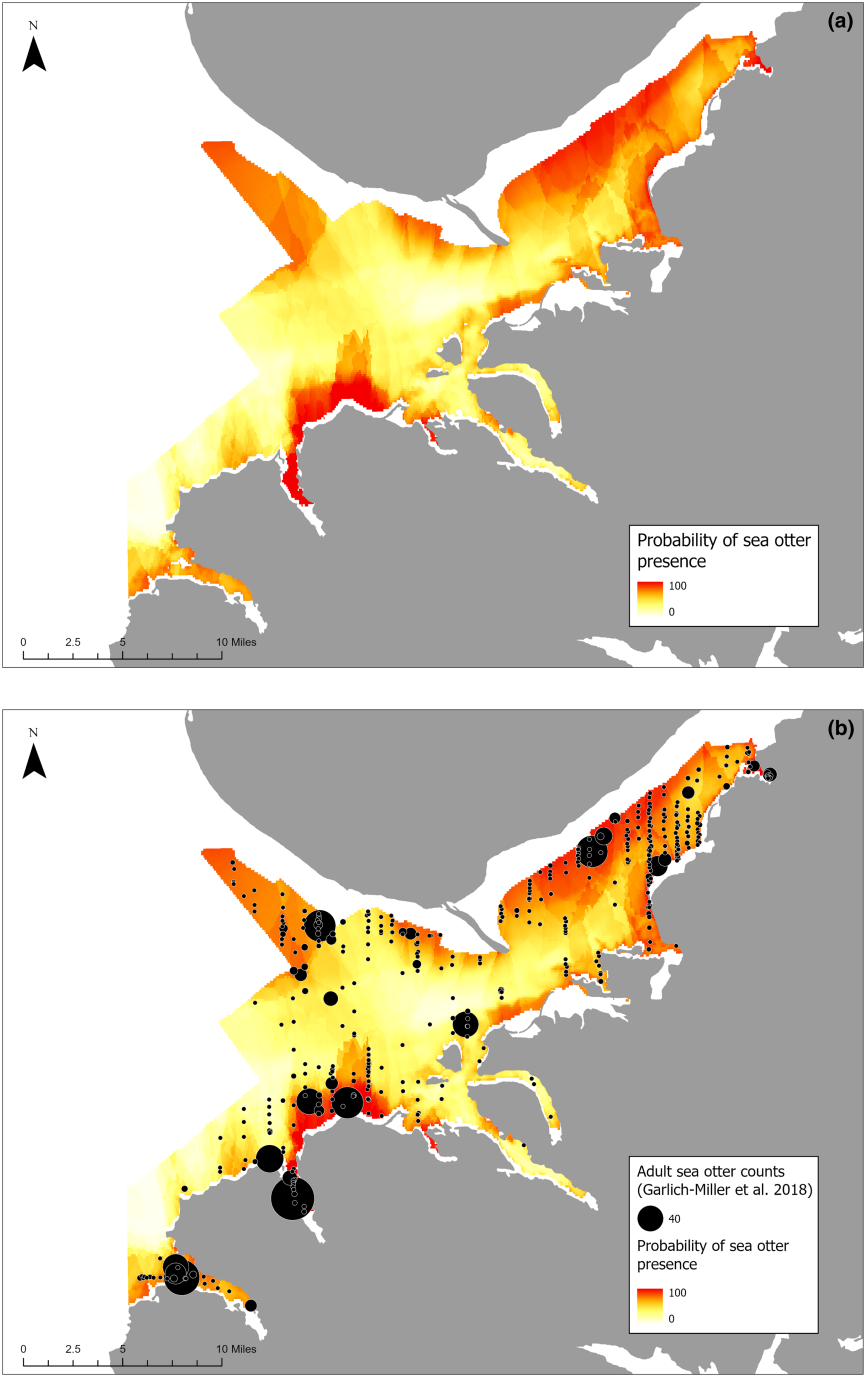
(a) Map of the probability of sea otter presence in Kachemak Bay based on MaxEnt model predictions. Probability ranges 0–100% from white to red. (b) Map of probability of sea otter presence in Kachemak Bay based on model predictions with sea otter survey results superimposed. Points represent adult sea otter observations and point size represents relative abundance with the point in the bottom legend representing 40 sea otters (Garlich‐Miller et al., 2018).

The empirical model was used to predict probability of sea otter occurrence throughout Kachemak Bay based on substrate, algae, and bathymetry (Figure 7a). These results reveal hotspots (i.e., high use areas) with high probability of sea otter occurrence in Kachemak Bay. Based on subtidal habitat associations, sea otters are likely to occur in the northwest, northeast, south‐southwest, and southwest areas of Kachemak Bay; however, there are variable hotspots throughout the bay (Figure 7a). These hotspots visually follow patterns of sea otter observations (Figure 7b) (Esslinger et al., 2021; Garlich‐Miller et al., 2018).

## 4 DISCUSSION

### 4.1 Model performance

This study leveraged open‐access data and demonstrated that sea otter species distribution modeling is feasible with temporally and spatially limited data, defined as having a short or no time series and course spatial resolution. The MaxEnt model was quantitatively evaluated to perform better than random using the methods described by Bohl et al. (2019). Results reported here confirm previously known sea otter habitat associations, such as a positive association with shallow depth (Bodkin et al., 2004; Coletti, 2006; Eisaguirre et al., 2021; Gilkinson et al., 2011; Williams et al., 2019; Yeates et al., 2007), and a positive association with understory kelp (Estes & Duggins, 1995; Foster & Schiel, 1988). Our analysis also supported novel relationships, such as a positive association with filamentous algae and a negative association with shell litter cover. Notably, areas within the study region that were predicted to have a higher probability of sea otter occurrence based on habitat associations also visually corresponded to areas with observations of greater sea otter abundance (Garlich‐Miller et al., 2018; Figure 7). This result would not be expected if the modeled habitat associations were not relevant to true sea otter distributions. Sea otter species distribution modeling, including variables, such as depth, rocky substrate, canopy kelp, and other environmental variables, has been conducted for California southern sea otters (*E. lutris nereis*) using a model that required continuous habitat data and multiple years of southern sea otter abundance survey data (Tinker et al., 2021). Such extensive spatial and temporal data are rarely available for modeling. Hence, our approach serves as a way forward for systems with more limited data.

In this exercise, model performance was evaluated to ensure that the model settings were the most appropriate. The final MaxEnt model that was selected had linear features and a regularization multiplier of one. A lower regularization multiplier corresponds to a weaker penalty on the model for complexity, resulting in a more complex framework (Merow et al., 2013). When comparing the AUC scores for both final candidate MaxEnt models, they both performed similarly in predictive accuracy with AUC_val_ = 0.681 for the sequentially selected model and AUC_val_ = 0.676 for the AICc selected model. However, the omission rate of 0.0996 for the sequentially selected model and 0.110 for the AICc selected model indicates a difference in overfitting. An omission rate higher than the given threshold (10%) indicates overfitting (Radosavljevic & Anderson, 2014). The sequentially selected model does not indicate overfitting, while the AICc selected model does. This comparison demonstrates the improved ability of the sequential method in limiting overfit models over the commonly used AICc method, which corroborates previous findings (Bohl et al., 2019; Kass et al., 2021; Radosavljevic & Anderson, 2014). The sequentially selected model is the model referred to hereafter. The model AUC_val_ score of 0.681 indicates prediction that deviates from random (AUC = 0.5) but is between “poor” and “fair” performance (Swets, 1988). While AUC can be further improved through the addition and omission of covariates (discussed further below), this performance is acceptable for a highly dimensional ecological model.

The results of the empirical to null model distribution comparison demonstrate that the discriminatory ability and the degree of overfitting of the empirical model are significantly better than the null model distribution at *α* = 0.05. This result conveys that the model can predict the probability of sea otter occurrence based on the observed locations better than a model based on randomly selected data and is less overfit than a model based on randomly selected data. Additional explanatory covariates, such as prey availability (Davis et al., 2019), distance from shore (Coletti, 2006), presence of canopy kelp (Tinker et al., 2021), and demography (Laidre et al., 2001; Tinker et al., 2008) may also increase the predictive power of the model, which would be demonstrated by an increase in AUC score above the 0.70 threshold of “fair” into the “good” range (Swets, 1988) and may further decrease the degree of overfitting by reducing complex relationships with less important covariates. However, this study demonstrates that the MaxEnt model produced here is quantitatively effective for species distribution modeling based on 2 years of open‐access environmental data and 1 year of species observation data and identified the importance of bathymetry, shell litter, and filamentous algal cover for predicting the probability of sea otter occurrence.

### 4.2 Habitat associations

Similar to other studies, bathymetry was found to be the most influential habitat attribute for predicting the probability of sea otter occurrence (see Bodkin et al., 2004; Coletti, 2006; Eisaguirre et al., 2021; Gilkinson et al., 2011; Williams et al., 2019; Yeates et al., 2007). Bathymetry was positively related with the probability of sea otter occurrence, such that sea otters are most likely to be present in shallower waters. Sea otters typically use shallower water depths (0–40 m), potentially due to a higher abundance of available prey compared to deeper waters (Bodkin et al., 2004; Gilkinson et al., 2011) and lower energetic costs of shallower dives (Yeates et al., 2007). Other studies have assumed that abundance and carrying capacity estimates can be applied to areas with similar habitat; however, similar habitat has previously been defined predominantly with depth alone (0–40 m; Laidre et al., 2002; Burn et al., 2003; Garlich‐Miller et al., 2018). Shallow depth may represent access to prey with minimal physiological diving stress and thermoregulatory costs (Yeates et al., 2007), but infaunal and epifaunal prey are variable in distribution and density across similar depths (Barber et al., 2012; Cates, 2022; Eggleston et al., 1992; Seitz et al., 2001; Sponaugle & Lawton, 1990; Turner et al., 1997). Hence, this may result in over‐ or under‐estimation of equilibrium density and abundance if habitat is more or less suitable in modeled areas based on variables that do not include prey availability.

Although sea otters forage on clams and deposit empty shells on the benthos, shell litter has not been examined by other habitat association studies. Here, shell litter was the second most important attribute in predicting the probability of sea otter occurrence. Shell litter had a negative relationship with probability of sea otter occurrence, such that an increase in percent cover of shell litter corresponded to a decrease in probability of sea otter occurrence. This relationship may indicate that areas with high shell litter cover no longer have an abundance of available bivalve prey. Areas with high shell litter cover may have limited remaining large live clams, thus causing sea otters to prey switch or forage in new locations, as has been observed when preferred prey stocks are depleted (Ostfeld, 1982).

Filamentous algae has not been considered in other sea otter habitat studies but was detected as the third most important attribute in predicting probability of sea otter presence in this study. Filamentous algae had a positive relationship with probability of sea otter occurrence, such that an increase in percent cover of filamentous algae corresponded to an increase in probability of sea otter occurrence. This association may be due to a positive feedback loop, where sea otters modify substrate and increase settlement of algal spores (Fletcher & Callow, 1992). Sea otters often feed in consolidated substrate habitat, where filamentous algae may recruit well and have low competition with larger/perennial algae (Stewart & Konar, 2012). Another hypothesis is that filamentous algae may be serving as a proxy for another factor that may be affecting sea otters more directly, such as prey abundance. Sea otters prey on herbivorous invertebrates (e.g., sea urchins) that may be feeding on filamentous algae (Dethier & Duggins, 1988; Vadas, 1977). In addition, filamentous algae may increase the amount of particulate organic carbon in the water column for filter‐feeding prey items (e.g., bivalves) to take up (Page, 1997; Riera & Richard, 1996). Additional covariates, such as invertebrate abundance and particulate organic carbon, would help distinguish the driving factor of the observed filamentous algal relationship but were not collected in this study.

Some, but not all, of the remaining covariates in the model only contributed minimally to predicting the probability of sea otter occurrence. Understory kelp was the fourth most influential attribute predicting sea otter occurrence, but only at 6.2% contribution, supporting findings from other studies of sea otters associating with understory kelp (Estes & Duggins, 1995; Foster & Schiel, 1988). Previous modeling studies have found canopy‐forming kelp associations with sea otters (Estes et al., 2010; Tinker et al., 2021). However, the current study only used non‐canopy‐forming algae, so algal results are not directly comparable. Of the remaining habitat attributes, gravel, pebble, and cobble only have minimal and negative relationships with probability of sea otter occurrence at 5.3, 3.4, and 2.3% contribution, respectively, and coralline algae has a minimal positive relationship with probability of sea otter presence at 2.9% contribution. Although percent contribution was <6% for these habitat attributes, this result demonstrates the prominence of habitat complexity in ecological relationships. Macroalgae, boulder, rugosity, and sand did not contribute to predicting probability of sea otter presence indicating that Kachemak Bay, Alaska, does not appear to function strictly as a rocky ecosystem based on substrate composition (Stewart et al., 2014).

Additional studies using habitat data have included substrate and distance to other known important features (e.g., distance to shore and distance to closest protected shoreline) and substrate as covariates in estimating density and distribution (Coletti, 2006; DeMaster et al., 1996; Eisaguirre et al., 2021; Laidre et al., 2002; Lu et al., 2020; Tinker et al., 2021; Williams et al., 2019). Specifically, distance to shore and distance to the closest protected shoreline have shown inverse relationships with sea otter density (Coletti, 2006). In addition, rocky, sandy, and mixed substrate types have been associated with varying degrees of sea otter carrying capacity (DeMaster et al., 1996; Laidre et al., 2001), though substrate, other than shell litter, was not a strong driving factor for predicting probability of sea otter occurrence in this study. Other sea otter distribution modeling studies have relied on population growth dynamics for modeling, which requires many consecutive years of sea otter surveys (Eisaguirre et al., 2021; Hale et al., 2022; Lu et al., 2020; Tinker et al., 2019; Williams et al., 2019).

Variation in results between this study and others may be due to survey method and data types. The use of drop cameras instead of multibeam sonar might lead to a discrepancy in scale. Drop camera data allow for a fine‐scale characterization of habitat and may provide benefits for studies emphasizing environmental complexity. In addition, leveraging technology, such as drop cameras, allows for increased survey extent without an increase in field effort, such as with in situ survey methods, that is, SCUBA diving, due to ease of use. Increasing the spatial extent of habitat data would broaden our range for predictions of animal distributions. The habitat associations in this study are based on a single summer season during 1 year of sea otter aerial abundance surveys, which is a snapshot in time for habitat use and does not capture seasonal or inter‐annual habitat use. Because the MaxEnt algorithm uses available space as background points for comparison, the resulting associations are still robust estimates of preferential habitat use. However, these associations are being drawn from data on both resting and foraging animals; these two behaviors might result in variable use of habitat types (Finerty et al., 2009). Tracking information would provide more detailed estimates of sea otter habitat use to correlate known behaviors and movement across space and time, though these data are challenging to collect for sea otters, especially in remote locations (Davis et al., 2019).

This study is a step forward in exploring additional habitat features that may be important for estimating sea otter carrying capacity in southcentral Alaska, as has been done in California with southern sea otters (Tinker et al., 2021). However, a component necessary for carrying capacity estimates is prey availability data, which influences equilibrium densities of sea otters (Davis et al., 2021; Dean et al., 2002). Despite large efforts to model carrying capacity in other regions, many studies (Eisaguirre et al., 2021; Hale et al., 2022; Lu et al., 2020; Tinker et al., 2019, 2021; Williams et al., 2019), including the one presented here, have not incorporated sea otter prey information directly, which is recommended for future modeling efforts (Davis et al., 2019). An important linkage is the association of subtidal habitat, specifically substrate and algal cover, with sea otter prey. Including estimates of available prey energy is also critical for accurately estimating equilibrium density of sea otters. Regions with stable sea otter populations have been found to increase in abundance after being considered standards for equilibrium densities, pointing to prey availability as a more reliable metric (Davis et al., 2021; Hale et al., 2019, 2022). We recommend combining a suite of known sea otter‐associated metrics in future studies to improve model AUC. Additional advancements in survey technology, including remote and autonomous survey platforms, may improve access to information in data‐limited systems. Much can be learned about the function of ecosystems by including physical habitat variables (Pittman, 2017).

Identifying habitat requirements is important for species management and conservation. As this study has shown, it is promising that modeling species distributions and habitat associations is possible in data‐limited ecosystems. Much is still unknown about our oceans and the ability to answer questions with open‐access data can help fill key information gaps. Beyond sea otters, this framework can be beneficial for species distribution modeling of other mobile marine megafauna. Specifically, understanding the habitat requirements of protected species and predicting distributions can mitigate future conflicts of resource competition or disturbance. The minimal data requirements of animal presence and dispersed habitat data over an area of interest can lead to significant gains in knowledge for management and conservation by using MaxEnt for species distribution modeling in data‐limited ecosystems.

## AUTHOR CONTRIBUTIONS

**Elizabeth L. Hasan:** Conceptualization (equal); data curation (lead); formal analysis (lead); funding acquisition (lead); investigation (equal); methodology (equal); writing – original draft (lead); writing – review and editing (equal). **Kristen B. Gorman:** Conceptualization (equal); investigation (equal); methodology (equal); writing – review and editing (equal). **Heather A. Coletti:** Conceptualization (equal); investigation (equal); methodology (equal); writing – review and editing (equal). **Brenda Konar:** Conceptualization (equal); funding acquisition (supporting); investigation (equal); methodology (equal); supervision (lead); writing – review and editing (equal).

## CONFLICT OF INTEREST STATEMENT

We have no competing interests to declare.

## Supporting information


Data S1.

## Data Availability

The habitat data that support the findings of this study are available in the Data S1 of this article.
